# Reprogramming tendon healing: a guide to novel molecular tools

**DOI:** 10.3389/fbioe.2024.1379773

**Published:** 2024-05-09

**Authors:** Carlos Julio Peniche Silva, Elizabeth R. Balmayor, Martijn van Griensven

**Affiliations:** ^1^ Cell Biology-Inspired Tissue Engineering, MERLN Institute for Technology-inspired Regenerative Medicine, Maastricht University, Maastricht, Netherlands; ^2^ Experimental Orthopaedics and Trauma Surgery, Department of Orthopaedic, Trauma, and Reconstructive Surgery, RWTH Aachen University Hospital, Aachen, Germany

**Keywords:** tendon, siRNA, miRNA, lncRNA, RNAi, mRNA silencing

## Abstract

Tendons are a frequent site of injury, which greatly impairs the movement and locomotion of patients. Regrettably, injuries at the tendon frequently require surgical intervention, which leads to a long path to recovery. Moreover, the healing of tendons often involves the formation of scar tissue at the site of injury with poor mechanical properties and prone to re-injury. Tissue engineering carries the promise of better and more effective solutions to the improper healing of tendons. Lately, the field of regenerative medicine has seen a significant increase in the focus on the potential use of non-coding RNAs (e.g., siRNAs, miRNAs, and lncRNAs) as molecular tools for tendon tissue engineering. This class of molecules is being investigated due to their ability to act as epigenetic regulators of gene expression and protein production. Thus, providing a molecular instrument to fine-tune, reprogram, and modulate the processes of tendon differentiation, healing, and regeneration. This review focuses particularly on the latest advances involving the use of siRNAs, miRNAs, and lncRNAs in tendon tissue engineering applications.

## 1 Introduction

Tendons are a crucial component of the musculoskeletal system, allowing for the movement and stabilization of joints. Tendons sustain tensile loads and dissipate the stress generated by muscle contraction and joint movement. The general tendon morphology consists of a highly specialized extracellular matrix made up of proteoglycans, a high content of water, and anisotropically aligned collagen fibers. Around 80% of the dry weight of tendons comes from collagen, which organizes into hierarchical structures of fibrillar networks aligned in the direction of loading (Buckley et al., 2013; Chartier et al., 2021).

The most abundant type of collagen found in tendons is the fibril-forming collagen type I. Collagen type I is organized into microfibrils and fibrils, granting tendons its natural mechanical durability and strength. Collagen type II and type III are also found in tendons but in lower amounts. Collagen type II is mostly concentrated at the tendon-to-bone insertion site (i.e., enthesis) while collagen type III is always associated with collagen type I. Collagen type III forms thinner fibrils than collagen type I. These fibrils are typically more disorganized and are mechanically weaker than those formed by collagen type I. However, they play a key role in the healing and pathogenesis of tendons. During the initial stages of healing, the content of randomly oriented collagen type III fibers increases at the wound site forming a fibrous scar tissue. Later, this tissue is replaced by a stronger and better-aligned network of collagen type I (Buckley et al., 2013; Chartier et al., 2021). Unfortunately, this remodeling phase often fails to completely regenerate the uninjured morphology of the tendon, and the collagen type III-rich scar tissue remains (Nguyen and Hsu, 2020; Shen et al., 2022). Thus, this weakens the tissue and increases the chances of recurrent rupture. The poor healing that is often seen after tendon injuries constitutes a challenge that tissue engineers working on tendon regeneration are trying to address by combining biomaterial design, cells, and bioactive molecules.

The field of biomaterials for tendon tissue engineering applications is extensive (Li et al., 2022; Xue et al., 2022; Huang et al., 2023). It comprises the use of natural and/or synthetic biomaterials in a wide range of combinations and designs to provide the best possible substitute to the native tissue during the process of healing while promoting *de novo* tissue regeneration. Some of the most used natural polymers for tendon regeneration applications are collagen, silk fibroin, chitosan, and fibrin (Dietrich et al., 2015; Yan et al., 2017; Sarıkaya and Gümüşderelioğlu, 2021). Natural polymers are praised for their biocompatibility and biodegradability. Alternatively, synthetic biomaterials such as poly-Ɛ-caprolactone, poly(lactic acid), poly(glycolic acid), or poly(lactic-co-glycolic acid) have also gained significant attention due to their tunable mechanical properties (Sensini et al., 2019; Kempfert et al., 2022; Uyanik et al., 2022).

The development of tendon mimetic constructs usually combines the design of structures that mimic the morphology found in healthy tendons and the use of mesenchymal stem cells (MSCs) or tendon progenitor stem cells (TPSCs). MSCs and TPSCs have the potential to respond to the morphological cues provided by the tendon mimetic structures to differentiate towards a tenogenic lineage. Thus, they can promote the formation of tissue-engineered tendon-like tissue (Font Tellado et al., 2017; Pardo et al., 2022).

In our consideration, the latest advances based on the strategies for the obtention of tendon-mimetic cell-laden constructs for tendon tissue engineering have been extensively reviewed (Lim et al., 2019; Ruiz-Alonso et al., 2021; Huang et al., 2023). Instead, the present review is inspired by the increasing evidence supporting the role of bioactive molecules as candidates to aid and promote tendon differentiation, regeneration, and healing. In particular, we will focus on the non-coding RNA-mediated transcriptional and post-transcriptional regulation of gene expression in the context of tendon healing and regeneration. More specifically, on the potential use of short interference RNA (siRNA), microRNA (miRNA), and long non-coding RNA (lncRNA), as molecular tools for reprograming or fine-tuning the processes of inflammation, scarring, and tissue regeneration in tendon healing.

## 2 Non-coding RNAs and tendon tissue engineering

Non-coding RNAs (ncRNA) are a heterogeneous group of RNA transcripts that do not translate into protein. Instead, they are implicated in a myriad of other cellular processes, most notably genome organization and regulation of gene expression (Kapranov et al., 2007; Nemeth et al., 2023). Over 70% of the human genome encodes for ncRNAs, and several classes of ncRNAs have been identified. This includes circular RNAs (circRNA), ribosomal RNAs (rRNAs), small nuclear RNAs (snRNAs), Piwi-interacting RNAs (piRNAs), siRNAs, lncRNAs, and miRNAs (Uszczynska-Ratajczak et al., 2018; Nemeth et al., 2023). The discovery of the mechanisms of RNA interference (RNAi) via ncRNAs as mediators of gene silencing, allowed for the development of novel therapeutic strategies to treat human diseases (Fire et al., 1998; Pal et al., 2005; Felekkis and Deltas, 2006; Oh and Park, 2009). This led to the first-in-human trial of an RNAi therapeutic in cancer patients via silencing of VEGF and kinesis spindle protein (KSP) (Tabernero et al., 2013).

Some of the best-studied ncRNAs are siRNAs, miRNAs, and lncRNAs. They are recognized as key regulators in many biological processes and have been associated with various human diseases. As such, a multitude of synthetic siRNA-based therapies as well as miRNA and lncRNA-based therapies are currently under investigation (Beg et al., 2017; Colpaert and Calore, 2019; Jin et al., 2021; Cerqueira et al., 2022; DiStefano and Gerhard, 2022; Iacomino, 2023).

For many years, researchers have focused on the potential role of ncRNAs in those human diseases that account for the highest mortality worldwide, including cancer, neurodegenerative diseases, and infectious diseases (Nemeth et al., 2023). However, more recently, tissue engineers have dived into the intricate world of ncRNA as a potential source of promising therapeutic tools that could lead to important breakthroughs in the fields of regenerative medicine and tissue engineering. Hereunder, we will summarize and discuss the latest advances in relationship with the potential use of lncRNA, siRNA, and miRNA-based therapy in tendon-tissue engineering.

### 2.1 siRNAs

Small interfering RNAs (siRNAs) are a class of double-stranded RNA molecules, typically between 21 and 23 nucleotides in length that play a crucial role in the regulation of gene expression. They owe their name to their ability to mediate in a process known as RNA interference (RNAi), a natural mechanism that controls the activity of genes by promoting the degradation of mRNA (Ipsaro and Joshua-Tor, 2015; Lam et al., 2015).siRNAs are the result of the processing of double-stranded RNA molecules (dsRNA) by the RNase III-like enzyme Dicer. These dsRNAs can be directly transcribed by the cells although they are often thought of as exogenous dsRNAs that might come from infecting pathogens as well as being artificially introduced into the cell via transfection vectors (Oh and Park, 2009). Once a dsRNA is processed into siRNAs, it can interact with the RNA-induced silencing complex (RISC) to target for degradation those mRNA molecules to which the siRNA guide strand is fully complementary. Thus allowing for a highly specific gene-silencing effect (Lam et al., 2015).

Such mechanism of action has been exploited to specifically target disease-related genes, most commonly in the context of illnesses like cancer as well as to fight infectious pathogens (Geisbert et al., 2006; Zamora et al., 2011; Sakurai et al., 2014; Chen Y. et al., 2015). Nevertheless, the field of regenerative medicine is rapidly expanding and recently has begun to explore novel tissue engineering applications based on RNAi.

Early studies involving siRNAs and tendons were mostly focused on the use of siRNA-mediated knockdowns to identify novel molecules relevant to the development, homeostasis, and normal function of tendons (Richardson et al., 2007; Tiwari et al., 2015; Gargano et al., 2021). Some examples include the identification of PIN1 (Peptidylprolyl Cis/Trans Isomerase, NIMA-Interacting 1) as a senescence inducer of tendon stem/progenitor cells (TSPC) (Chen L. et al., 2015). Additionally, RNAi knockdown of the transcription factor protein P65 revealed that P65 promoted fibrogenic and proinflammatory activity in tendon fibroblasts (Chen et al., 2017). Similarly, siRNA-targeting of the activated transcription factor 6 (ATF-6) revealed an antifibrotic role for ATF-6 in TGβ-1 pretreated fibroblasts from the Achilles tendon in a rat model (Yao et al., 2019). Moreover, by performing a tendon cell-specific RNAi screening, Tiwari et al. reported 19 novel molecules with enzymatic function or known to be involved in transcription activity, cell adhesion, protein folding, and intracellular transport functions in the context of the myotendinous junction of *Drosophila* (Tiwari et al., 2015).

The previous examples not only contributed to revealing the fundamental biological functions of the molecules in question but also pointed to potential therapeutic applications for the siRNA-mediated modulation of tendon fibrosis and healing. Thus, RNAi has been increasingly explored as a therapeutic candidate to improve tendon healing. For instance, Liao et al. reported the use of a collagen III-targeting siRNA to suppress the expression of collagen type III in tenocytes cultured in the presence of TGFβ-1. Thus, showing a proof of concept where a siRNA-based approach could potentially serve as treatment for the prevention of fibrosis by regulating collagen type III production in tendon-related disorders (Liao et al., 2020).

Another study aimed at the improvement of tendon healing, investigated the role of the small collagen fibrils in tendon repair, more specifically collagen type V. Collagen type V is typically increased during healing and plays an important role in fibrillogenesis (Lu et al., 2011). Lu et al. demonstrated that *COLV*-siRNA-engineered tenocytes displayed better tendon regeneration capabilities by promoting the formation of larger collagen fibrils achieving improved tendon contour and morphology. However, this study concluded that the ratio between collagen type V and collagen type I should be carefully monitored as the full knockdown of *COLV* hinders the formation of normal collagen fibrils. Hence, RNAi could be used to modulate the expression of *COLV* to achieve the desired balance of collagen type I and type V production necessary for the effective regeneration of healthy tendon tissue while minimizing the occurrence of fibrosis (Lu et al., 2011).

The combination of siRNA-based therapeutic approaches with the use of biomaterials for tendon tissue engineering has proven to enhance the potency of RNAi in tendon healing applications. Cai et al. developed a self-healing hydrogel encapsulating *SMAD3*-siRNA as an antiadhesion barrier to prevent tendon fibrosis and improve tendon healing *in vivo*. The self-healing capabilities of the hydrogel allowed for an attenuated inflammation of the injured tendon as a consequence of the reduction of the shear stress between the hydrogel-wrapped injured tendon and the peritendinous tissue. Moreover, the *SMAD3*-siRNA reduced the expression levels of *SMAD3*, leading to a decrease in the activation of the *TGF-β1/SMAD3* pathway and the consequent reduction in fibroblast proliferation and collagen type III production (Cai et al., 2022).

Despite the growing interest in the potential tissue-engineering applications for siRNA and RNAi technology in general, practical limitations to their use in the clinic are still to be overcome (Ali Zaidi et al., 2023). siRNAs can be degraded by endosomal nucleases or remain trapped indefinitely in non-functional stress granules or other cytoplasmatic bodies, which would greatly affect their efficacy (LeCher et al., 2017; Wang et al., 2021). Additionally, siRNA entrapment can also occur in the extracellular space, where proteins from the serum could form a non-functional protein-siRNA complex, thus, hindering the siRNA therapeutic effect (Ali Zaidi et al., 2023).

### 2.2 miRNAs

MicroRNAs, also known as miRNAs, are naturally occurring, short-non codding RNAs usually between 19 and 25 nucleotides in length. They are typically transcribed by the RNA polymerase II and, even when some miRNAs are individually produced from separate transcription units, they can also be produced as clusters of different miRNAs out from larger transcript-encoding miRNAs (Denli et al., 2004). Directly after transcription, pri-miRNAs are obtained, which will be later processed into pre-miRNAs. These are stem-loop structures that are exported from the cell nuclei to the cytoplasm where the terminal loop is removed by the enzyme Dicer to create a mature miRNA duplex (Bartel, 2004; Carthew and Sontheimer, 2009). Similarly to siRNA, miRNAs are effectors of the RISC complex and mediate the posttranscriptional regulation of a myriad of genes (Carthew and Sontheimer, 2009).

One of the most distinctive features of miRNAs is their ability to interact with hundreds of different mRNA sequences. This is believed to be due to the fact that miRNAs can target mRNA sequences to which they are not perfectly complementary. Furthermore, the degree of miRNA-mRNA complementarity is a crucial determining factor of their regulatory mechanism (Bartel, 2004; Carthew and Sontheimer, 2009). Perfect complementarity often leads to the degradation of the mRNA by the RISC complex while partial complementarity can sequester the mRNA without achieving cleavage of the mRNA strand. In the latter scenario, the recycling of the miRNA to the RISC complex can be delayed, and the miRNA-mRNA interaction accelerates the decay of the miRNA strand, as was demonstrated by a kinetic analysis of the fate of miRNAs after target regulation by Baccarini et al. (Carthew and Sontheimer, 2009; Baccarini et al., 2011).

As miRNAs are endogenous to the cell, miRNA-based therapies can rely either on miRNA replacement or inhibition. Typically, miRNA replacement is done through the use of miRNA mimics, while miRNA inhibition is achieved with the use of antagomirs or miRNA inhibitors (Gori et al., 2015).

In tendon tissue engineering, a plethora of miRNAs have been and continue to be investigated for their potential regulation over relevant tenogenic pathways. The miR-29 family is one of the best studied in the context of tendon healing (Millar et al., 2015; Liu et al., 2021). miR-29a, a member of this family, is known to regulate the production of collagen type III in tendon fibroblasts. This inspired Watts et al. who used intralesional injections of miRNA-29a in an equine tendon model to achieve improved tendon healing by reducing the expression of *COLIII* while increasing the expression of *COLI*. Likewise, miR-29b is reported to regulate collagen production by interacting with the *SMAD3/TGF-β1* pathway. According to a study by Chen et al., the overexpression of miR-29b in the Achilles tendons of rats improved tendon healing and reduced scar formation after surgery (Chen et al., 2014). Once again, the regulation of the *SMAD3/TGF-β1* pathway by ncRNAs is a target for potential therapeutic approaches to achieve tendon healing. Furthermore, miRNA-based regulation of collagen production in tendon cells has been reported via alternative pathways. miR-124-3p was found to inhibit *EGR1*, which is known to activate the expression of the tendon markers *MKX*, *SCX*, and *COLI* (Guerquin et al., 2013). The inhibition of EGR1 by the overexpression of miR-124-3p in hTDSCs (tendon-derived stem cells) prevented tendon differentiation whilst the inhibition of miR-124-3p promoted the opposite effect (Wang et al., 2016). Thus, suggesting that miR-124-3p is a promising therapeutic target for tendon injury and healing.

In a recent study by our group, fibrosis-related miRNA profiling in a rodent patellar injury model allowed for the identification of dysregulated miRNAs at different time points after injury (Peniche Silva et al., 2023). A total of 13 miRNAs known or predicted to interact with important tenogenic pathways were identified to be dysregulated upon tendon-to-bone enthesis injury. Among them, the previously mentioned miR-124-3p was found upregulated while *EGR1* was downregulated. Additionally, miR-16-5p and miR-133-3p were strongly upregulated in the fibrotic portion of the tendon side of the enthesis 10 days after injury. Interestingly, both miRNAs are known for their anti-fibrotic potential and are reported to inhibit myofibroblasts activation by regulating *SMAD3* and *COLI* respectively (Wei et al., 2019; Yao et al., 2020). Hence, their upregulation at the tendon side of the enthesis after injury suggested an antifibrotic role for these miRNAs in tendon healing. This highlights the relevance of miR16-5p and miR-133-3p as therapeutic candidates to aid tendon healing and regeneration.

In another study involving miRNA profiling, Plachel et al. profiled miRNAs in samples from sera and biopsy samples from the supraspinatus and subscapularis tendons from patients suffering from degenerative rotator cuff tears (RCT), chronic rotator cuff tendinopathy, and healthy patients. They reported at least six circulating miRNAs (i.e., miR-18, miR-19a, miR19b, miR-25, mR-93, and miR192) that were downregulated both in sera and biopsy samples in patients from degenerative RCT when normalized against healthy controls. Furthermore, another six miRNAs were dysregulated in both chronic tendinopathy and degenerative RCT: miR-30-5p, miR-140-3p, miR-210-3p, miR-222-3p, miR-324-3p, miR-425-5p (Plachel et al., 2020). Such data contribute to the identification and establishment of miRNA signatures not only as therapeutic tools but also as diagnostic and prognostic tools for degenerative and chronic rotator cuff tendinopathies.

Inflammation is well known to play a major role in tendon healing and scar formation (Arvind and Huang, 2021; Chartier et al., 2021). Moreover, miR-205 has been found implicated in the secretion of inflammatory factors and the amplification of the NF-kβ-induced inflammatory response in cancer cells (Yeh et al., 2016). However, it has been reported that the inhibition of miR-205 in rat tenocytes from the Achilles tendon leads to an increase in the expression of the anti-inflammatory effector MECP2 (methylated binding protein 2). Furthermore, the inhibition of miR-205 improved tenocyte proliferation and migration and increased the expression of *COLI*, *COLIII*, *SCX,* and *TNC*. Hence, suggesting a tenogenic effect for the inhibition of miR-205.

The RNAi mechanisms of siRNA and miRNA replacement therapy are in many ways similar. They both are based on the administration of synthetic siRNAs or miRNAs to achieve gene silencing. However, miRNAs have the potential to interact with a multitude of different pathways while siRNAs are specifically designed to target one gene of interest. This highlights an important difference that sets these classes of molecules apart, in particular when considering aspects of their sequence design and therapeutic approach. Additionally, miRNA-based therapy comprehends the inhibition of miRNAs by means of miRNA inhibitors, an approach that has no equivalent in the work with siRNAs. Nevertheless, these types of small RNA molecules face similar challenges that hinder their applications in the clinic such as poor *in vivo* stability, the need for efficient transfection vectors, and off-target effects (Lam et al., 2015).

### 2.3 LncRNAs

LncRNAs are non-coding transcripts longer than 200 nucleotides, although such length cut-off appears to be somewhat arbitrary (Ponting et al., 2009; Cao, 2014). When first discovered, lncRNAs were thought to be non-functional. However, there is now plenty of evidence for the roles of lncRNAs as genomic regulators as well as regulators of transcription and translation, interacting either directly with DNA, and mRNA or acting as miRNA sponges, thus, affecting cell identity, fate, and function (Cao, 2014; DiStefano and Gerhard, 2022; Nemeth et al., 2023).

RNA-sequencing has allowed for lncRNA profiling, hence, facilitating the identification of differentially expressed lncRNAs in specific settings. In a conjoint analysis of lncRNA and mRNA expression in the context of a RCT, Ge et al. identified 419 lncRNAs and 1,542 mRNAs that were differentially expressed in patients with RCT in comparison with the expression in normal tendon. Furthermore, competitive endogenous RNA network analysis based on those results revealed interactions between 139 lncRNA, 126 mRNA and 35 miRNAs, most of which were related to the citrate cycle, p53 signaling, and the renin-angiotensin system. Additionally, they found differentially expressed genes involved in VEGF signaling, which is in line with the changes in vascularity typically observed in RCT. Thus, providing insights into the potential lncRNA-mRNA-mediated mechanism underlying tendon pathology (Ge et al., 2020).

Among the lncRNAs described to be dysregulated in tendinopathy, lncRNA X-inactive specific transcript (lncRNA XIST) has been found highly expressed in relationship with tendon injury (Peffers et al., 2015). Nevertheless, contrasting functions have been described for XIST. In ligament fibroblasts, XIST promotes osteogenic differentiation via the lncRNA XIST/miR-302a-3p/USP8 axis (Yuan et al., 2021). While cancer research acknowledges XIST as a cancer-promoting gene due to its association with tumor occurrence and development via targeting of the tumor-suppressing miR-34a-5p and miR-137 (Wang et al., 2017; Sun et al., 2018). However, in the context of tendon injury in mice models, the overexpression of XIST in populations at high risk of tendon injury was linked to the decreased expression of miR-26-5p and the increased expression of cyclooxygenase 2 (COX2), with the consequent increase in fibroblast proliferation, collagen production, and the occurrence of tendon adhesion. Indicating a role for lncRNA XIST targeted miR-26-5p in the healing of tendon injury (Chen et al., 2022).

Other reports highlight the tenogenic role of lncRNAs. Such is the case of the lncRNA H19, which has been described to significantly accelerate the TGF-β1-induced tenogenic differentiation *in vitro* and accelerate tendon healing in mouse tendon defect models *in vivo* (Lu et al., 2017). H19 promotes tenogenesis by directly targeting miR-29b-3p. As mentioned before, miR-29b-3p has the potential to suppress the expression of *TGF-β1* and collagen type I (Chen et al., 2014; Lu et al., 2017). Thus, the TGF-β1/H19/miR-29b-3p regulatory loop could be the target of new strategies for treating tendon injuries. Similarly, lncRNA MALAT1 has been shown to promote tendon healing in rat models of tendinopathy by regulating the miR-378a-3p/MAPK1 axis. MiR-378a is a biomarker for tendon injury and its over-expression is associated with decreased *COL1A1, SCX, MKX, MMP3* and other tendon markers. Thus, MALAT1-mediated regulation of miR-378a-3p could be another potential target of molecular therapies to aid tendon healing.

## 3 Future perspectives

Non-coding RNAs are increasingly present in the development of novel tissue engineering approaches to treat tendon injuries. As molecular tools to modulate gene expression and protein production, they hold the promise to lead the field of regenerative medicine toward a more personalized kind of medicine. Tailoring the ncRNA-based strategies to individual patient profiles may improve the efficacy of the treatments. Moreover, even when the possibility for off-target effects is still a concern when working with ncRNAs, they offer superior specificity to the traditional gene manipulation methods (Kohn et al., 2023). Additionally, they can be integrated into various biomaterials and scaffolds to achieve enhanced regeneration capacity.

**FIGURE 1:**
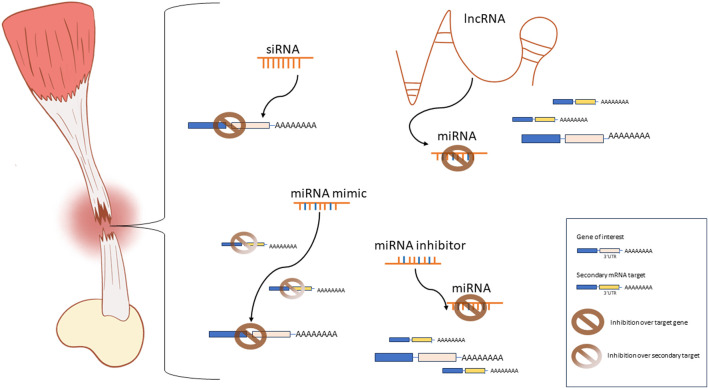
Schematic representation of the most commonly used ncRNA-based approaches for tendon tissue engineering applications. siRNAs are designed to specifically interact with one mRNA sequence to achieve inhibition of gene expression. miRNA mimics inhibit one specific mRNA target while interactions with secondary targets and alternative pathways are possible. miRNA inhibitors prevent the miRNA-mediated repression of the mRNA target by inhibiting the activity of the endogenous miRNAs. lncRNAs are used as miRNA sponges to inhibit the miRNA-mediated repression of a target gene.

In our consideration, there is enough evidence of the potential benefits associated with the use of ncRNAs in regenerative medicine applications to justify the increasing interest in researching this class of molecules, their mechanisms of action, and potential applications in tendon tissue engineering. Nevertheless, siRNAs, miRNAs, and lncRNAs exhibit individual strengths and limitations that should be carefully considered when investigating their potential applications (Figure 1). The RNAi mechanisms of siRNA and miRNA are similar. However, siRNA can be specifically designed to target one mRNA sequence. Alternatively, miRNAs can interact with many distinct biological pathways and many pathways can regulate one specific miRNA. This is both a curse and a blessing, and extensive research is still required to fully understand the implications of dysregulating miRNAs in a tissue-specific manner. Moreover, antagomirs or miRNA inhibitors provide a valuable tool to research the effects of the suppression or knockdown of miRNAs. Similarly, lncRNAs can directly interact with miRNAs, acting as miRNA inhibitors thus restoring the function of the miRNA-targeted mRNA. However, lncRNAs exhibit a wide range of targets beyond miRNAs. They can interact with DNA, proteins, or mRNA. Additionally, they can be found in either the nucleus, affecting chromatin structure, or in the cytoplasm, modulating transcriptional and post-transcriptional processes (Jin et al., 2021; Winkle et al., 2021). Compared to siRNAs and miRNAs, lncRNA are functionally very complex and their function is reported to be context dependent. Hence, the understanding of the precise mechanisms and specific function of individual lncRNAs in each tissue is an ongoing challenge. For the moment, the focus on lncRNA in tendon tissue engineering applications seems to be more or less limited to their regulation over miRNAs. Future studies may unveil new applications addressed to aid tendon healing and regeneration.

## 4 Conclusion

Tendons play a fundamental role in movement and locomotion. Thus, injuries at the tendon can greatly impair the quality of life of patients and represent a significant societal and economic burden. Moreover, patients suffering from tendon injury undergo a long and often painful path to recovery. Advances in the field of tendon tissue engineering are expected to lead to better tissue healing with less scar formation and superior recapitulation of the native tendon morphology and function. ncRNAs offer a set of powerful tools to fine-tune at the molecular levels processes of cell differentiation, proliferation, matrix deposition, and tissue remodeling that could greatly aid tissue regeneration and healing. However, siRNAs, miRNAs, and lncRNA have only recently emerged as molecular candidates for tendon tissue engineering applications. Thus, extensive research is still required to fully harness their potential for better healing of tendons.
